# The Effects of ARCS Motivational Instruction in Physical Education on Learning Cognition and the Health-Related Physical Fitness of Students

**DOI:** 10.3389/fpsyg.2022.786178

**Published:** 2022-06-06

**Authors:** Xi Luo, Liu Liu, Jingjing Li

**Affiliations:** ^1^School of Physical Education, Sichuan University, Chengdu, China; ^2^School of Gymnastics, University of Electronic Science and Technology of China, Chengdu, China

**Keywords:** ARCS motivation model, instruction in physical education, learning cognition, health related physical fitness, value component

## Abstract

The environment in metropolitan regions along with other factors such as changes in lifestyle and academic pressure can result in students reducing the time they spend pursuing outdoor activities. An increase in sedentary lifestyles and lack of physical activity seriously threaten the health of students, due to reduced physical fitness. To solve this issue, cultivating exercise habits should commence from childhood. Physical education in schools is the best time to cultivate the development of a wholesome body and mind in students. Students need to have high flexibility, strong learning ability, and regular exercise in schools for their future physical and mental development, the establishment of an exercise regimen, and the cultivation of motor skills. For this study, university students in Sichuan Province were chosen as research samples, and 500 copies of a questionnaire were distributed among them. After removing invalid and incomplete questionnaires, 375 were deemed valid, a retrieval rate of 75%. The research results demonstrated significant positive correlations between (1) the Attention, Relevance, Confidence, and Satisfaction (ARCS) motivational instruction in physical education and learning cognition, (2) learning cognition and health-related physical fitness, and (3) ARCS motivational instruction in physical education and health-related physical fitness. The study results suggest that a good exercise regimen boosts students' self-confidence, increases their motivation to participate in physical activities, enhances their health-related physical fitness, and cultivates habits to engage in lifelong physical activity.

## Introduction

The World Health Organization (WHO) cautions that inadequate physical activity is the fourth major hazard to global mortality. Annually, more than 2 million human deaths worldwide are related to insufficient physical activity. The proportion of the population with inadequate physical activity is increasing in many countries (Bechter et al., 2021). Barring sportspersons in various countries, exercise is often not a priority. Competition is fierce in the workplace, and only those who work fast and perform better at work stand out in this competitive environment. This results in a lack of physical movement (Wu et al., 2022). The environment in metropolitan regions, changes in lifestyle, and effects of academic pressure result in students largely reducing their time for outdoor activity after school. Besides, an increase in sedentary lifestyles and lack of physical activity have become primary threats to students' health, resulting in a marked decline in their physical fitness (Tong et al., 2021). Therefore, physical education (PE) in schools should prepare students to adapt to modern life, understand their bodies, and cultivate health management habits (Wu et al., 2021). The inculcation of good health and physical fitness behaviors in students should be the primary objective of health and physical education instructions (Li et al., 2021).

Advances in technology and changes in lifestyle have decreased physical activity and exercise in students, resulting in poor physical fitness and abnormal body postures (Batistič et al., 2021). In addition to an unbalanced diet, preferences for high-calorie food and insufficient exercise are the factors contributing to childhood obesity. Good exercise habits in adults start during childhood. Physical education in schools plays a crucial role in cultivating a wholesome body and mind in students. The exercise regimen at schools, particularly for flexibility and stamina, deeply impacts future physical and mental development in students along with an understanding of the need to exercise, the formation of exercise habits, and the cultivation of motor skills. The development of exercise habits during the student phase is critical; the cultivation of regular exercise habits from childhood could enhance development, promote health and physical fitness, and ensure they continue to exercise after growing up. Physical education in schools, therefore, is important. It becomes important for PE teachers in schools to make their students see exercise as fun, which also facilitates health-related physical fitness and enhances their quality of life. There are advantages to traditional teaching models that future education reforms should take into account while making improvements. While providing physical education instructions it is necessary to observe the students' learning effectiveness in physical fitness courses (Wu et al., 2020a). The curriculum design should present exercise as fun to continue motivating students' physical activities, provide development and learning opportunities, allow students to enjoy the learning process, and maintain good physical fitness standards. Motivation is also an important indicator of individual learning efficiency (Small and Gluck, 1994). The ARCS motivation model, which refers to Attention, Relevance, Confidence, and Satisfaction, is a systematic teaching method with an instructional design model to motivate students' continuous learning needs. The model is based on motivation theory and is practical (Keller, 1983). Zhu and Burrow (2022) maintained that ARCS could reinforce systematic instructional design to encourage learners' participation and interaction. Additionally, this model provides a theoretical basis and practical application. In addition to the viewpoint of behaviorism, the theoretical foundation also emphasizes individual cognition, expectation, and value. The ARCS model also aligns with the contemporary educational thought of openness, freedom, and personal value. For these reasons, the impact of ARCS motivational instruction in physical education on students' learning cognition and health-related physical fitness are discussed in this study. The study also aimed to explore how the ARCS model can help students acquire successful experience in exercise, build their self-confidence, increase their motivation to participate in physical activity, enhance their health-related physical fitness, and cultivate lifelong habits in students to engage in physical activity.

## Literature Review and Hypothesis

Chin et al. (2018) mention four factors in the ARCS motivation model that closely impact teaching. When teachers have to include the ARCS model in their teaching to develop a benign loop in the students' learning cognition; the lack of any part was said to reduce the entire learning cognition effect. Chang and Hwang (2018) pointed out Keller's emphasis on the diagnostic nature and prescriptive function of ARCS. They explained that instructors can provide systematic instructional strategies to compensate for insufficient motivation and improve learning cognition of students who lack these four conditions. Wu (2018) argued that teachers should understand and use strategies such as the ARCS model in their instructional design to develop and produce materials that attract students and motivate them to learn, as it is the key factor in determining teaching success and students' learning cognition outcome. Accordingly, the following hypotheses were tested in this study.

**H1**: ARCS motivational instruction in physical education has significant correlations with learning cognition.
**H1-0**: ARCS motivational instruction in physical education negatively correlates with learning cognition.
**H1-1**: ARCS motivational instruction in physical education positively correlates with learning cognition.

Lin et al. (2018) studied the effectiveness and relationship between motor skill learning and learners' motivations (ARCS learning motivation) in information integrated instruction in physical education for pupils. An experimental group showed a significant positive correlation between learning cognition and motor skill learning. Learning cognition presented the strongest predictability of motor skill learning (Wu et al., 2020b). Similarly, Chen and Lin (2018) studied the effects of the ARCS motivation model on G3 pupils' motor skills, health-related physical fitness, and learning cognition and found significant positive correlations between the experimental group's learning cognition and their motor skills, health-related physical fitness, attention, relevance cognition, self-confidence, and satisfaction. With the intervention of the ARCS motivation model, Deublein et al. (2018) found that students in the experimental group and the control group appeared to have notable differences in overall learning motivation, attention, learning cognition, self-confidence, and satisfaction in learning motivation as well as significant differences on overall skill learning effect and health-related physical fitness. Accordingly, the following hypothesis was tested in this study.

**H2**: Learning cognition has significant correlations with health-related physical fitness.
**H2-0**: Learning cognition has significant negative correlations with health-related physical fitness.
**H2-1**: Learning cognition has significant positive correlations with health-related physical fitness.

Chen et al. (2018) studied the effect of exercise duration on elementary school pupils' health-related physical fitness. They discovered that increasing the teaching hours for PE had a significant impact on students' cardiorespiratory capacity, flexibility, muscle strength, and muscular endurance in health-related physical fitness. In research on the effect of walk-run activity on elementary school pupils' health-related physical fitness, Ibáñez and Delgado-Kloos (2018) discovered that the activity could enhance the students' physical fitness, and female students showed remarkable differences in more items than male students did. Li and Keller (2018) studied the impact of different types of new-style calisthenics training on elementary school children's health-related physical fitness. The results showed that students who trained in new-style calisthenics made noticeable progress on health-related physical fitness, especially in the 800-m walk run; male and female students who trained three times per week showed notable progress on the 1-min bent-knee sit-up and 800-m walk-run. Therefore, it was suggested that training three times per week was best for enhancing elementary school children's physical fitness. Accordingly, the following hypothesis was established in this study.

**H3:** ARCS motivational instruction in physical education has significant correlations with health-related physical fitness.
**H3-0:** ARCS motivational instruction in physical education has significant negative correlations with health-related physical fitness.
**H3-1:** ARCS motivational instruction in physical education has significant positive correlations with health-related physical fitness.

## Methodology

### Conceptual Structure of This Study

**Figure 1:**
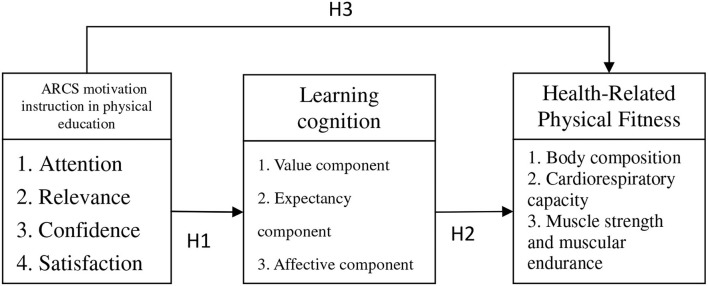
Conceptual structure.

Summing up the above literature review, the conceptual structure of the research (Figure 1) lays out the relationship between ARCS motivational instruction in physical education, learning cognition, and health-related physical fitness.

### Operational Definition

1. ARCS motivational instruction in physical education

Referring to Hong et al. (2019), ARCS motivational instruction contains four dimensions.

1. Attention: Inducing interest in students and sustaining their attention are the first priorities in this model. Capturing students' attention is the first element of learning.
2. Relevance: The second element of the model is to make the students realize that what they are learning is relevant. The design needs to be customized to the students' characteristics, knowledge, and cultural background to enhance students' interests in learning.
3. Confidence: Confidence involves an individual's belief in completing their work. When students believe that they can successfully learn new courses or complete new work, it demonstrates higher learning motivation.
4. Satisfaction: Satisfaction is students' evaluation of the learning results. Personal satisfaction is a key factor in remaining motivated.

(2) Learning cognition

Based on the study by Sung et al. (2019), three cognition components of value, expectancy, and affection have been adopted in this study to determine students' learning process.

1. Value component: students' perceived importance, value, and belief in the learning activity.
2. Expectancy component: students' belief in their ability to learn and the expectation to achieve learning.
3. Affective component: feelings and emotional responses of students to their personal ability to learn and the result.

(3) Health-related physical fitness

Referring to Kao et al. (2019), health-related physical fitness in this study contains four dimensions.

1. Body composition: It refers to the ratio or content of various structural components in the body.
2. Cardiorespiratory capacity: It refers to the ability of the heart to transmit blood and oxygen to the entire body.
3. Muscle strength and muscular endurance: Muscle strength refers to the ability of muscles or muscle groups to develop the maximal strength to withstand resistance within a total activity.
4. Flexibility: It refers to the maximal range of activity, i.e., the activity range of joints and periarticular ligaments and muscle extension abilities.

### Research Sample

Five hundred copies of the questionnaire were distributed among college students in Sichuan Province who served as the research sample. After removing invalid and incomplete copies, 375 responses were deemed valid, with a valid retrieval rate of 75%.

## Results

### Factor Analysis

The results of the factor analysis are shown in Table 1. The factor analysis of the scale of ARCS motivational instruction in physical education extracted the following four factors: “attention” (eigenvalue = 2.755, α = 0.82), “relevance” (eigenvalue = 2.163, α = 0.88), “confidence” (eigenvalue = 1.836, α = 0.83), and “satisfaction” (eigenvalue = 1.442, α = 0.80). The cumulative covariance was 71.694%. The factor analysis for learning cognition scale extracted the following three factors: “value component” (eigenvalue = 3.623, α = 0.84), “expectancy component” (eigenvalue = 2.514, α = 0.86), and “affective component” (eigenvalue = 2.287, α = 0.85). The cumulative covariance was 73.281%.

**Table 1:** Factor analysis.

**Variable**	**Dimension**	**Eigenvalue**	**α**	**Cumulative variance explained**
ARCS motivational instruction in physical education	Attention	2.755	0.82	71.694
	Relevance	2.163	0.88	
	Confidence	1.836	0.83	
	Satisfaction	1.442	0.80	
Learning cognition	value component	3.623	0.84	73.281
	Expectancy component	2.514	0.86	
	Affective component	2.287	0.85	

### Correlation Analysis

Table 2 shows notable correlations between ARCS motivational instruction in physical education, learning cognition, and health-related physical fitness. These results support H1, H2, and H3.

**Table 2:** Correlation analysis.

**Research dimension**	**α**	**ARCS motivational instruction in physical education**	**Learning cognition**	**Health-related physical fitness**
ARCS motivational instruction in physical education	0.84			
Learning cognition	0.85	0.26^**^		
Health-related physical fitness	0.92	0.31^**^	0.23^**^	

** *Stands for p < 0.01*.

### LISREL Evaluation Indicator

Linear structural relation (LISREL) combines factor analysis and path analysis in traditional statistics and adds simultaneous equations in econometrics. It is a research tool to calculate multiple factors and multiple casual paths simultaneously. The goodness-of-fit of the model could be evaluated from preliminary fit criteria, overall model fit, and fit of the internal structure of the model.

The research results are organized as below. The preliminary fit, internal fit, and overall fit of the model are explained.

From the complete model analysis results, as shown in Table 3, four factors in ARCS motivational instruction in physical education (attention, relevance, confidence, and satisfaction) significantly explain ARCS motivational instruction in physical education (*t* > 1.96, *p* < 0.05); three factors in learning cognition (value component, expectancy component, and affective component) remarkably explain learning cognition (*t* > 1.96, *p* < 0.05); four factors in health-related physical fitness (body composition, cardiorespiratory capacity, muscle strength, and muscular endurance, and flexibility) notably explain health-related physical fitness (*t* > 1.96, *p* < 0.05). Therefore, the overall research model shows a good preliminary fit.

**Table 3:** Overall linear structural model analysis result.

**Evaluation item**	**Parameter/evaluation standard**		**Result**
Preliminary fit	ARCS motivational instruction in physical education	Attention	0.726^**^
		Relevance	0.738^**^
		Confidence	0.750^**^
		Satisfaction	0.707^*^
	Learning cognition	Value component	0.746^**^
		Expectancy component	0.718^*^
		Affective component	0.763^**^
	Health-related physical fitness	Body composition	0.755^**^
		Cardiorespiratory capacity	0.783^**^
		Muscle strength and muscular endurance	0.777^**^
		Flexibility	0.769^**^

** *Stands for p <0.01*,

* * Stands for p <0.05*.

In terms of internal fit, ARCS motivational instruction in physical education reveals positive and significant correlations with learning cognition (0.342, *p* < 0.01). Learning cognition appears positive and has remarkable correlations with health-related physical fitness (0.296, *p* < 0.01). In addition, ARCS motivational instruction in physical education shows positive correlations with health-related physical fitness (0.388, *p* < 0.01). H1, H2, and H3 are therefore supported.

Regarding the overall model fit, the overall model fit standards, χ^2^/Df = 1.763, which is smaller than standard 3 and RMR is 0.006, show proper results of χ^2^/DF and RMR. Furthermore, the chi-square test is sensitive to sample size and it is not suitable for directly judging the fit. However, the overall model fit standards, GFI = 0.987 and AGFI = 0.944, are higher than the standard 0.9 (the closer GFI and AGFI to 1 revealing the better model fit). This model, therefore, presents better goodness-of-fit.

## Discussion

The ARCS motivation model can match various subjects and materials to develop distinct teaching strategies for promoting learning motivation. Many studies affirm that applying the ARCS motivation model to subject teaching in different fields presents a positive value to the teaching and learning environment, either as guidance or the development of teaching strategies. For instruction in physical education, the students' attention is first captured according to the ARCS motivation model. Their curiosity about the curriculum is aroused by asking questions so that students are fully involved in the learning activity and get confident in completing their learning. With intrinsic and extrinsic encouragement, the learning desire and self-satisfaction are sustained. In other words, it is expected that students experience fun and comfort, are inspired by exercise, and present good physical fitness through PE in schools. By reinforcing positive ideas about sports, the students are prompted to keep exercising and encourage the sports culture. ARCS motivational instruction in physical education could enhance students' learning cognition in terms of attention, relevance, confidence, and satisfaction to further promote learning effectiveness (Harlen and Crick, 2003) as well as improve body mass index, flexibility, muscle strength, and muscular endurance, and cardiorespiratory capacity in students' health-related physical fitness (Ryan and Deci, 2000). Exercise has significant positive effects on flexibility, muscle strength and muscular endurance, and cardiorespiratory capacity (Ormrod, 2003), especially abdominal muscle strength and muscular endurance, which is important to maintain body posture. With bad abdominal muscle strength and muscular endurance, the pelvis would not be suspended in a normal position and might appear tilted, further affecting health. Good muscle strength and muscular endurance strengthen the ligaments and tendons and reduce fatigue or injury during physical activity (Stipek, 1995). For this reason, the promotion of a good sports atmosphere and the design of physical activity, as well as the intervention of physical fitness games, could make physical education fun and lively to spark students' learning interests and active learning of various motor skills. As a result, students could cultivate the good habit of lifelong exercise. It is the urgent responsibility of PE teachers. The research results confirm that ARCS motivational instruction in physical education could effectively promote students' learning cognition and health-related physical fitness, capture students' attention in PE, establish relevant cognition, and build self-confidence and self-satisfaction. ARCS motivational instruction in physical education could also enhance students' body mass index. When activity time and frequency are increased in the future, the effect might be more obvious.

## Conclusion

Learning motivation promotes learning behavior and explains why an individual is willing to sacrifice other activities to participate in certain activities. Inducing motivation in students is the first step in the teaching process, which enables students to show interest and induce existing knowledge, and prepares them to learn new things. Teachers urgently need skills and strategies to enhance students' learning motivation in current teaching sites. It is an important factor for success in teaching and students' learning outcomes. Mandigo and Corlett (2010) point out that the instructional objectives of the PE curriculum are for students to learn correct sports concepts and motion skills and cultivate the habit of lifelong sports and a healthy body and mind. However, a high level of learning motivation is essential for students' continuous learning in PE to achieve learning objectives. Students, therefore, acquire learning satisfaction and experience in PE courses by participating in PE classes and PE-related activities. This reinforces their intrinsic learning motivation by enjoying such a special experience (Kirk and Kinchin, 2003). By stressing teaching activity, building a good learning atmosphere, and utilizing complete equipment and facilities to create a quality learning environment, PE teachers could enhance students' learning effectiveness and self-confidence while establishing the effectiveness of promotion and development of the instruction (Taplin, 2019).

## Data Availability Statement

The original contributions presented in the study are included in the article/supplementary material, further inquiries can be directed to the corresponding author.

## Ethics Statement

The studies involving human participants were reviewed and approved by the Ethics Committee of the Sichuan University. The participants provided their written informed consent to participate in this study.

## Author Contributions

XL performed the initial analyses and wrote the manuscript. LL and JL assisted in the data collection and data analysis. All authors revised and approved the submitted version of the manuscript.

## Funding

This research is supported by the Fundamental Research Funds for the Central Universities in Sichuan University.

## Conflict of Interest

The authors declare that the research was conducted in the absence of any commercial or financial relationships that could be construed as a potential conflict of interest.

## Publisher's Note

All claims expressed in this article are solely those of the authors and do not necessarily represent those of their affiliated organizations, or those of the publisher, the editors and the reviewers. Any product that may be evaluated in this article, or claim that may be made by its manufacturer, is not guaranteed or endorsed by the publisher.
